# Commentary: Preoperative neutrophil to lymphocyte ratio predicts complications after esophageal resection that can be used as inclusion criteria for enhanced recovery after surgery

**DOI:** 10.3389/fsurg.2022.1016196

**Published:** 2022-10-21

**Authors:** Cheng Shen, Qiang Pu, Guowei Che

**Affiliations:** ^1^ Department of Thoracic Surgery, West-China Hospital, Sichuan University, Chengdu, China; ^2^ Department of Lung Cancer Center, West-China Hospital, Sichuan University, Chengdu, China

**Keywords:** enhanced recovery after surgery, neutrophil to lymphocyte ratio (NLR), surgery, complication, inflammatory

**A Commentary on**
Preoperative neutrophil to lymphocyte ratio predicts complications after esophageal resection that can be used as inclusion criteria for enhanced recovery after surgery By Shi BW, Xu L, Gong CX, Yang F, Han YD, Chen HZ and Li CG. (2022) Front Surg. 9:897716. doi: 10.3389/fsurg.2022.897716

## Introduction

It was a pleasure to go through the manuscript by Shi et al. (1), published in *Frontiers in Surgery*, which focuses on how the preoperative neutrophil to lymphocyte ratio (NLR) can be used as inclusion criteria for enhanced recovery after surgery (ERAS) in complications after esophageal resection, as he brings up excellent discussion topics.

## Discussion

ERAS is a multidisciplinary collaboration, multimodal coexistence, and evidence-based approach, which improves the nursing status of surgical patients by adopting a full range of optimized solutions during the perioperative period. This improves the quality of postoperative recovery and shortens the recovery time for the patient (2). The lower the incidence of postoperative complications, the faster the postoperative recovery in a patient undergoing surgery (3). Early studies on ERAS were performed in patients undergoing colorectal surgery. With the ERAS concept being recognized by most surgeons, it is now used in many different surgical specialties (4).

It is well known that surgical trauma can lead to significant inflammatory and immune response disorders in the body. The goal of ERAS is to promote the physiological stability of the patient's body and promote the rapid healing of wounds, modulating the systemic inflammatory response (SIR) (5). SIR markers such as NLR are the most clinically valuable. NLR can be used as a detection content as it contains the key information of serum biomarkers, and its role is closely related to the complications that occur during the early recovery of patients (6). However, we found few studies about NLR and ERAS after conducting a systematic literature review. We performed this research to summarize the connection between NLR and ERAS in different surgery.

In the research done by Shi et al*.* (1), 171 patients who underwent an esophagectomy were included in the study. All the patients included in the study completed three blood tests, and the time points for blood collection were the week before surgery, the day of surgery, and the first day after surgery. The blood test consisted of a white blood cell count and neutrophil, lymphocyte, and albumin quantification. In the study, they found that an elevated NLR value before surgery was closely related to the occurrence of postoperative complications and was an independent risk factor in the patient after the esophagectomy. Therefore, combined with the test results, it is speculated that NLR can more accurately predict the occurrence of postoperative complications of esophageal cancer. In this way, clinicians can better deal with the complications. However, the complications and recovery time of esophageal resection vary when considering different surgical approaches, tumor locations, and whether or not minimally invasive therapy is used. We need more clinical studies to refine the findings, including different surgical modalities (robotic, minimally invasive, and thoracotomy) and different surgical scopes to further clarify whether NLR applies to all patients in different states.

NLR was also used in a study of head and neck surgery with the ERAS program (7). The preoperative mean NLR values in the two groups were similar (the ERAS group and the control group). Compared with the control group, the NLR values of the ERAS intervention group showed a trend of significant decrease on postoperative day 1 and postoperative day 3 (*p* < 0.01). Such experimental results show that the systemic inflammatory response in the ERAS group was significantly inhibited by the perioperative ERAS protocol. In the study by Peng et al. (8), the ERAS protocol was used in gynecological surgery. A total number of 130 surgical patients with gynecologic tumors were included in this study. Among them, there were 65 patients in the ERAS program group and 65 patients in the control group. By comparing the results of preoperative NLR in the two groups, there was no significant difference between them. However, patients in the ERAS protocol group showed a trend of significantly lower NLR after the surgery compared with the control group. Such experimental results demonstrate that the ERAS procedure can have an inhibitory effect on possible postoperative SIR, which may also be a new marker for the perioperative evaluation in gynecological oncology in the future. However, other studies have shown contrasting results. Although the experimental group was implemented in the perioperative period according to the ERAS protocol, there was no statistically significant difference in postoperative NLR levels compared with the control group in the research done by both Zhou et al. (9) and Jaloun et al*.* (10).

Previous studies have found that in patients with cancer, the higher the level of NLR expression, the greater the possibility of lymph node metastasis. However, it remains to be confirmed whether there is a high degree of relevance in the implementation of the ERAS program. In the future, we need to conduct a prospective multicenter randomized controlled trial with a larger sample size to clarify the advantages of NLR and to further prove the guiding role of the ERAS protocol in the perioperative period of different types of tumors.
